# Methionine and vitamin B‐complex ameliorate antitubercular drugs‐induced toxicity in exposed patients

**DOI:** 10.1002/prp2.360

**Published:** 2017-09-21

**Authors:** Kennedy I. Amagon, Olufunsho Awodele, Abidemi J. Akindele

**Affiliations:** ^1^ Department of Pharmacology Therapeutics and Toxicology College of Medicine University of Lagos Lagos Nigeria; ^2^ Department of Pharmacology and Toxicology Faculty of Pharmaceutical Sciences University of Jos Jos Nigeria

**Keywords:** Antioxidants, biomarkers, drug toxicity, methionine, modulation, tuberculosis

## Abstract

Tuberculosis therapy utilizes drugs that while effective cause treatment‐related toxicity. Modulation of antitubercular drugs‐induced toxicity by methionine and vitamin B‐complex in patients was evaluated. 285 treatment‐naïve tuberculosis patients at the Chest Clinics of Infectious Diseases Hospital, Yaba and General Hospital, Lagos in Lagos, Nigeria was prospectively recruited and allotted into test (antitubercular medicines, methionine and vitamin B‐complex) and control groups (antitubercular medicines). Data on adverse drug reactions and blood samples were collected at initiation, 2 months and 6 months, and then analyzed. Red blood cells and packed cell volume were significantly higher (*P *<* *0.05) in the test group compared to control at 6 months of therapy. At the end of 2 months, results showed a significant decrease (*P *<* *0.001) in aspartate aminotransferase, alkaline phosphatase, alanine aminotransferase, urea, creatinine and total bilirubin in the test group compared to control. Reduced glutathione and superoxide dismutase were significantly increased (*P *<* *0.001) and malondialdehyde significantly decreased (*P *<* *0.001) in the test versus control groups at the end of 2 and 6 months. Adverse drug reactions were significantly lower (*P *<* *0.001) in the test group (32.4%) compared to control group (56.2%), with 1 death. Hepatotoxicity was significantly higher (*P *=* *0.026) in control (6.9%), compared to test group (0%). Alcohol and cigarette smoking were significantly (*P *=* *0.019 and *P *=* *0.027) associated with the occurrence of adverse drug reactions. Methionine and vitamin B‐complex modulated hepatic, renal, hematological, antioxidant indices and adverse effects in patients administered antitubercular medicines. Such interventions can enhance compliance and better treatment outcomes in tuberculosis patients.

**Abbreviations**
DILI: drug‐induced liver injury
INH: isoniazid
MDA: malondialdehyde
PZA: pyrazinamide
RIF: rifampin
ROS: reactive oxygen species
RUCAM: Roussel Uclaf Causality assessment method
SAMe: S‐adenosyl‐L‐methionine
TB: tuberculosis

## Introduction

Tuberculosis (TB), an infectious disease caused by *Mycobacterium tuberculosis*, is a single leading cause of death from any single infectious agent and a major global public health problem worldwide (Mohajan 2015). Isoniazid (INH), rifampin (RIF), pyrazinamide (PZA), and ethambutol (EMB) are first‐line drugs used for tuberculosis therapy in adults for an initial 2 months (intensive phase), then a continuation phase of 4 months involving INH and RIF (Huiri et al. 2017). Despite the effectiveness of this treatment regimen, treatment‐related toxicity manifesting as hepatotoxicity, skin reactions, and gastrointestinal disturbances have been reported (Tostmann et al. 2008).

Reactive Oxygen Species (ROS) are produced in the presence of diseases or drugs, resulting in lipid peroxidation and oxidative stress (Zhai et al. 2008). Thus, a reduction in lipid peroxidation in tissue and increase in superoxide dismutase, catalase and glutathione activities would help to maintain cell integrity and control the increase in markers of oxidative stress (Hamza and Al‐Harbi 2015). Elevated hepatic, renal, hematological and antioxidant indices are important biomarkers in confirming toxicity and extent of organ damage.

The liver is implicated in drug‐induced toxicity as it plays an important role in the metabolism and excretion of many drugs, including antituberculosis drugs. Drug‐induced liver injury (DILI) causes acute and chronic disease, though the link between drugs and preexisting liver disease is complex, more so when symptoms develop in patients with liver disease during treatment (Teschke and Danan 2016). This problem is evident as many physicians attending to chronic liver disease patients are faced with determining if drug therapy is a risk that increases their patient's chances for drug‐induced liver injury (Teschke and Danan 2017). To properly establish cause and effect, it is appropriate to conduct an assessment of causality, using various criteria and methods, such as the Roussel Uclaf Causality Assessment Method (RUCAM) (Teschke and Danan 2016).

Drug‐induced toxicity can also cause clinical adverse effects, which can occur either among antitubercular medicines or between antitubercular medicines and other medicines (Farazi et al. 2014). This may extend or modify treatment, and cause drug resistance leading to treatment failure (Kaona et al. 2004) and death. Factors like age, sex, race, other drugs, breastfeeding and pregnancy (Alomar 2014) can increase the occurrence of adverse drug reactions. The very young and elderly people are more at risk of developing adverse drug reactions (ADRs) than other age groups (Pretorius et al. 2013), while the female gender has a higher risk of developing adverse drug reactions than males (Rademaker 2001).

The severity of antitubercular drug‐induced toxicity has led to the idea to use other drugs, which when co‐administered may prevent or significantly reduce toxicity. Methionine is an antioxidant that protects glutathione, the major antioxidant in human cells that protect against free radicals and toxic compounds. Vitamin B complex contains Vitamin B1 and B6, which have been reported to possess antioxidative properties (Hellmann and Mooney 2010; Alvarado and Navarro 2016). This study evaluated the modulatory effect of co‐administration of methionine and vitamin B‐complex on antitubercular drugs‐induced toxicity in tuberculosis patients.

## Materials and Methods

This prospective study was conducted amongst 285 treatment‐naïve tuberculosis patients at the Chest clinics of Mainland Hospital, Yaba and General Hospital, Lagos in Lagos, Nigeria, after ethical approval and informed consent were obtained. Participants were allotted into the test (co‐administered antitubercular medicines with methionine and vitamin B‐complex) and control groups (administered antitubercular medicines only). Test group participants were placed on 4 tablets of methionine daily (250 mg each) via the oral route and 2 tablets of vitamin B‐complex orally daily for 6 months, representing the period of TB treatment. This was in addition to the combined antitubercular medicine regimen (all patients).

Blood samples were collected at initiation of treatment, at 2 months and 6 months, then hematological, biochemical and antioxidant parameters were analyzed according to standard protocols. Data on patients’ demographics and reported adverse drug reactions was also collected prospectively.

Data entry, coding, cleaning and analysis was done using SPSS version 20.0. Descriptive statistics was summarized using frequency, proportions, measures of central tendency and dispersion. Bivariate analysis such as chi‐square test was used to investigate the association between adverse drug reaction and selected variables. Logistic regression was further used to determine the factors that may be significantly associated with adverse drug reactions by the patients. Comparisons between the baseline and follow‐up data were performed, using the Mann Whitney *U* test. All tests were carried out at 5% level of significance.

## Results

### Demographic characteristics of research participants

Most of the participants were males (71.28%) versus females (28.72%) in the present study. Participants were aged between 18 to 65 years, with majority (36.84%) aged 31–40 years and 34.39% were aged 18–30. Majority was married (53.19%), singles made up 37.94% of participants, while widows/widowers were the least (3.55%) (Table 1). Study participants with a secondary school certificate were more (50.18%), than those with tertiary education (25.96%).

**Table 1:** Demographic characteristics of research subjects

Characteristics	Group	All patients *n* (%)	Control group *n* (%)	Test group *n* (%)	*P*‐value
Sex	Male	201 (71.28)	92 (45.77)	109 (54.23)	0.137
	Female	81 (28.72)	45 (55.56)	36 (44.44)	
Age (years)	18–30	98 (34.39)	49 (50.00)	49 (50.00)	0.008^a^
	31–40	105 (36.84)	39 (37.14)	66 (62.86)	
	41–50	44 (15.44)	23 (52.27)	21 (47.73)	
	50+	38 (13.33)	26 (68.42)	12 (31.58)	
Marital status	Single	107 (37.94)	52 (48.6)	55 (51.40)	0.582
	Married	150 (53.19)	71 (47.33)	79 (52.67)	
	Divorced	15 (5.32)	7 (46.67)	8 (53.33)	
	Widow/widower	10 (3.55)	7 (70.00)	3 (30.00)	
Highest education	NFE/Primary	68 (23.86)	34 (50.00)	34 (50.00)	0.810
	Secondary	143 (50.18)	66 (46.15)	77 (53.85)	
	Tertiary	74 (25.96)	37 (50.00)	37 (50.00)	

a Chi‐square test (significant). NFE, no formal education.

### Effect of methionine and vitamin B‐complex on hepatic parameters in control and test groups at 2 and 6 months of antituberculosis drug treatment

At the end of 2 months of TB treatment, ALT, AST, ALP and total bilirubin were significantly (*P *<* *0.001) lower in test group participants (Median: 8.4, 22.1, 64.7 and 3.1) compared to participants in the control group (Median: 74.95, 56.25, 85.4 and 8.25) (Table 2).

**Table 2:** Effect of methionine and vitamin B‐complex on hepatic parameters in control and test groups at baseline, 2 and 6 months of antituberculosis drug treatment

	Parameter	Median (IQR) level Control (a)	Median (IQR) level Test (b)	*P*‐value
Baseline	AST (U/L)	22.80 (IQR: 15.25–32.10)	35.00 (IQR: 25.25–49.60)	<0.0001^a^
	ALT (U/L)	9.90 (IQR: 5.7–16.83)	14.80 (IQR: 7.40–27.10)	0.008^a^
	ALP (U/L)	65.60 (IQR: 54.85–90.58)	82.70 (IQR: 68.80–99.55)	0.002^a^
	T. Bilirubin (mmol/L)	4.75 (IQR: 3.35–6.65)	5.90 (IQR: 4.50–8.35)	0.01^a^
Month 2	AST (U/L)	56.25 (IQR: 47.20–63.95)	22.1 (IQR: 15.10–32.80)	<0.001^a^
	ALT (U/L)	74.95 (IQR: 65.38–89.03)	8.4 (IQR: 4.70–12.30)	<0.001^a^
	ALP (U/L)	85.4 (IQR: 80.35–102.50)	64.7 (IQR: 51.70–88.60)	<0.001^a^
	T. Bilirubin (mmol/L)	8.25 (IQR: 5.48–15.08)	3.1 (IQR: 2.50–4.00)	<0.001^a^
Month 6	AST (U/L)	30.1 (IQR: 19.60–35.50)	25.45 (IQR: 16.68–32.73)	0.276
	ALT (U/L)	17.2 (IQR: 5.30–70.10)	8.45 (IQR: 4.70–30.75)	0.183
	ALP (U/L)	71.35 (IQR: 56.28–83.03)	68.5 (IQR: 58.30–113.00)	0.370
	T. Bilirubin (mmol/L)	4.3 (IQR: 3.25–6.03)	4.2 (IQR: 3.60–5.30)	0.910

a Mann Whitney *U* test (Significant); IQR, Interquartile range.

ALT, AST, ALP, and total bilirubin were not significant (*P *>* *0.05) in the test group, relative to the control group participants at the end of 6 months of tuberculosis therapy (Table 2).

### Change in hepatic function parameters from baseline at 2 and 3 months of antituberculosis treatment

A significant difference (*P *<* *0.001) was observed in changes in AST, ALT, ALP and total bilirubin from baseline at 2 months between control and test group participants (Table 3). No difference was observed between these same parameters at 6 months compared to baseline. (Table 3).

**Table 3:** Change in hepatic function parameters from baseline at 2 and 6 months of antituberculosis treatment

	Control group Median (IQR) (2 or 6 months value Minus baseline value)	Test group Median (IQR) (2 or 6 months value Minus baseline value)	*P*‐value
Month 2			
AST (U/L)	33.45 (18.73–42.88)	−12.6 (−30.2 to 4.7)	<0.001^a^
ALT (U/L)	58.75 (48.68–73.98)	−12.5 (−17.6 to 6.5)	<0.001^a^
ALP (U/L)	14.1 (−2.8 to 35.88)	−15.1 (−27.2 to 4.1)	<0.001^a^
T_Bil (mmol/L)	3.2 (0–10.1)	−2.6 (−3.5 to 1.5)	<0.001^a^
Month 6			
AST (U/L)	4.75 (−11.93−16.33)	−4.7 (−33.5 to 8.9)	0.313
ALT (U/L)	−0.5 (−10.6 to 25.4)	10.8 (−11.6 to 55.3)	0.531
ALP (U/L)	8.3 (−30.3–25.43)	−2.2 (−52.6 to 17.9)	0.454
T_Bil (mmol/L)	0.15 (−2.78 to 1.3)	−1.1 (−3.7 to 0.5)	0.918

A positive median value of hepatic parameter shows an increase from baseline value, while a negative value indicate a decrease from baseline value.

a Mann Whitney *U* test (Significant); IQR, Interquartile range.

### Effect of methionine and vitamin B‐complex on renal parameters between test and control groups at 2 and 6 months of anti‐TB therapy

Creatinine and urea levels (Median: 63.13 and 2.6) were significantly (*P *<* *0.001) lower and albumin (Median: 37.1) significantly (*P *=* *0.096) lower in the test participants, relative to control group participants at the end of 2 months of TB treatment (Table 4). Total protein was not significant (*P *>* *0.05) in the test versus control group.

**Table 4:** Effect of methionine and vitamin B‐complex on renal parameters between test and control groups at baseline, 2 and 6 months of anti‐TB therapy

	Parameter	Median (IQR) Control (a)	Median (IQR) Test (b)	*P* value a versus. b
Baseline	Creatinine (mmol/L)	65.50 (IQR: 54.58–78.14)	74.31 (IQR: 63.76–88.38)	0.003^a^
	Urea (mmol/L)	3.20 (IQR: 2.50–4.10)	3.60 (IQR: 2.60–5.25)	0.068
	ALB (g/L)	32.80 (IQR: 28.90–36.25)	34.7 (IQR: 30.85–39.73)	0.150
	T.Protein	74.60 (IQR: 64.85–79.35)	77.30 (IQR: 71.58–81.73)	0.014^a^
Month 2	Creatinine (mmol/L)	94.25 (IQR: 81.53–136.24)	63.13 (IQR: 50.40–73.13)	<0.001^a^
	Urea (mmol/L)	7.65 (IQR: 5.25–8.95)	2.6 (IQR: 2.30–3.60)	<0.001^a^
	Alb (g/L)	35 (IQR: 31.60–38.95)	37.1 (IQR: 29.00–41.70)	0.096^a^
	T. Protein (g/L)	74.30 (IQR: 70.48–79.75)	74.7 (IQR: 65.80–82.10)	0.105
Month 6	Creatinine (mmol/L)	64.65 (IQR: 58.91–88.91)	62.84 (IQR: 58.13–81.37)	0.734
	Urea (mmol/L)	3.25 (IQR: 2.88–3.95)	3.2 (IQR: 2.60–4.10)	0.688
	Alb (g/L)	34.9 (IQR: 30.08–40.98)	36.5 (IQR: 33.10–40.60)	0.423
	T. Protein (g/L)	74.7 (IQR: 68.85–79.65)	71.2 (IQR: 69.80–80.10)	0.721

a Mann Whitney *U* test (Significant); IQR, Interquartile range.

There was no difference in levels of creatinine, urea, total protein and albumin in the test versus control group participants at the end of 6 months (Table 4).

### Change in renal function parameters from baseline at 2 and 6 months of antituberculosis treatment

A significant difference was observed in changes in creatinine and urea from baseline at 2 and 6 months between control and test group participants (Table 5). No difference was observed between albumin and total protein in both test and control participants at 2 and 6 months compared to baseline (Table 5).

**Table 5:** Change in renal function parameters from baseline at 2 and 6 months of antituberculosis treatment

	Control group (2 or 6 months value Minus baseline value)	Test group (2 or 6 months value Minus baseline value)	*P* value
Month 2			
Creatinine (mmol/L)	26.16 (12.75–74.83)	−22.34 (−33.5 to 2.88)	<0.001^a^
Urea (mmol/L)	4.4 (2.25–5.45)	−1.5 (−3.4 to 0.8)	<0.001^a^
ALB (g/L)	1.4 (−5.35 to 5.8)	1.8 (−10.2 to 10)	0.650
T. protein (g/L)	2.45 (−6.38 to 13.03)	0.2 (−13.3 to 8.6)	0.536
Month 6			
Creatinine (mmol/L)	1.65 (−13.99 to 15.42)	−7.58 (−28.7 to 4.53)	0.051^a^
Urea (mmol/L)	0.4 (−0.58 to 0.95)	−2 (−3.1 to 0.2)	0.01^a^
ALB (g/L)	0 (−6.15 to 6.28)	−0.5 (−6.3 to 7.2)	0.875
T. protein (g/L)	−0.35 (−9.2 to 5.78)	−3.2 (−10.3 to 2.6)	0.986

a Mann Whitney *U* test (Significant); IQR, Interquartile range.

A positive median value of hepatic parameter shows an increase from baseline value, while a negative value indicates a decrease from baseline value.

### Effect of methionine and vitamin B‐complex on antioxidant indices at 2 and 6 months of antitubercular therapy

**Figure 1:**
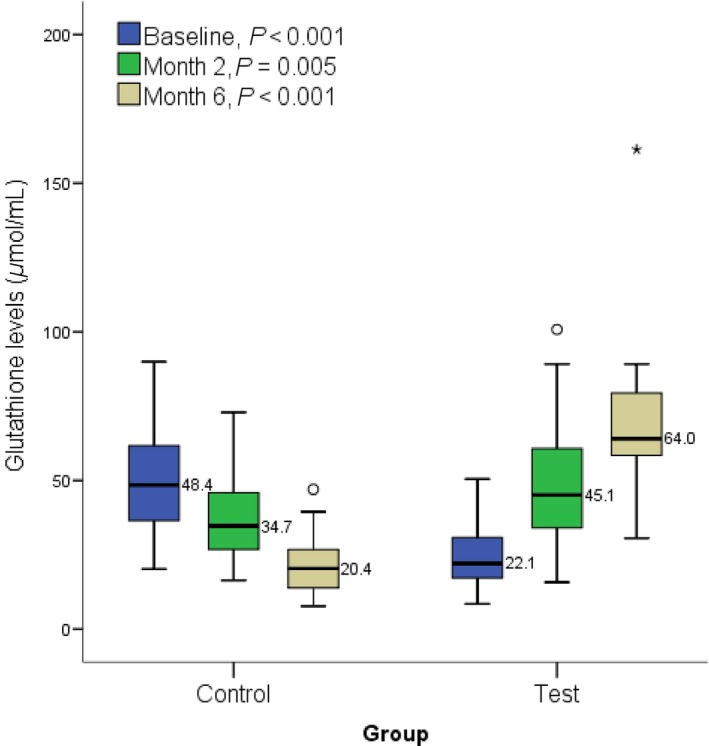
Showing effect of methionine and vitamin B‐complex on reduced glutathione levels at baseline, 2 and 6 months of TB treatment. Small circle “o” indicate Outliers or “out” values, while star “*” represent far out” or “Extreme values”.

**Figure 2:**
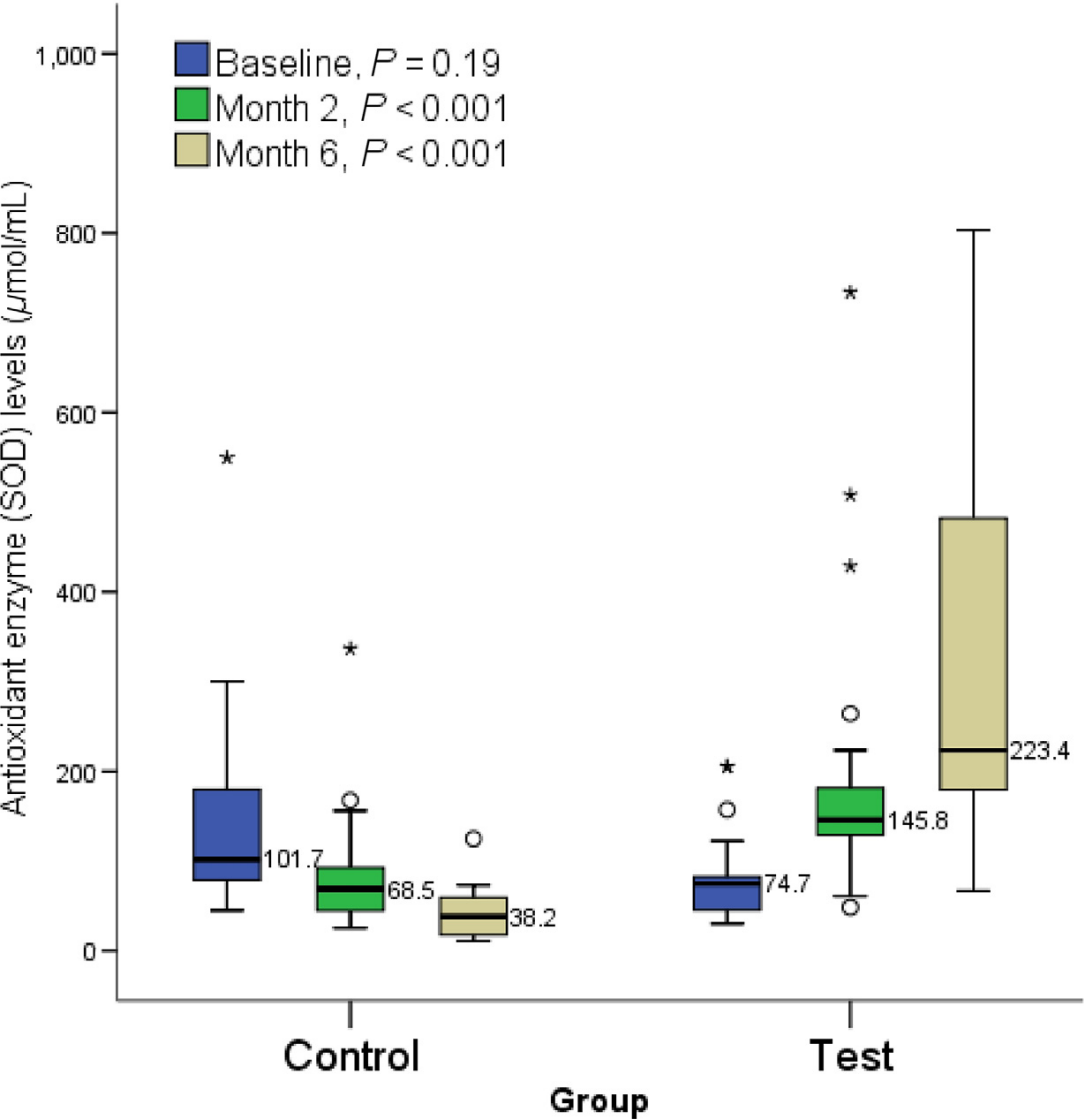
Showing effect of methionine and vitamin B‐complex on superoxide dismutase level at baseline, 2 and 6 months of TB treatment. Small circle “o” indicate Outliers or “out” values, while star “*” represent far out” or “Extreme values”

**Figure 3:**
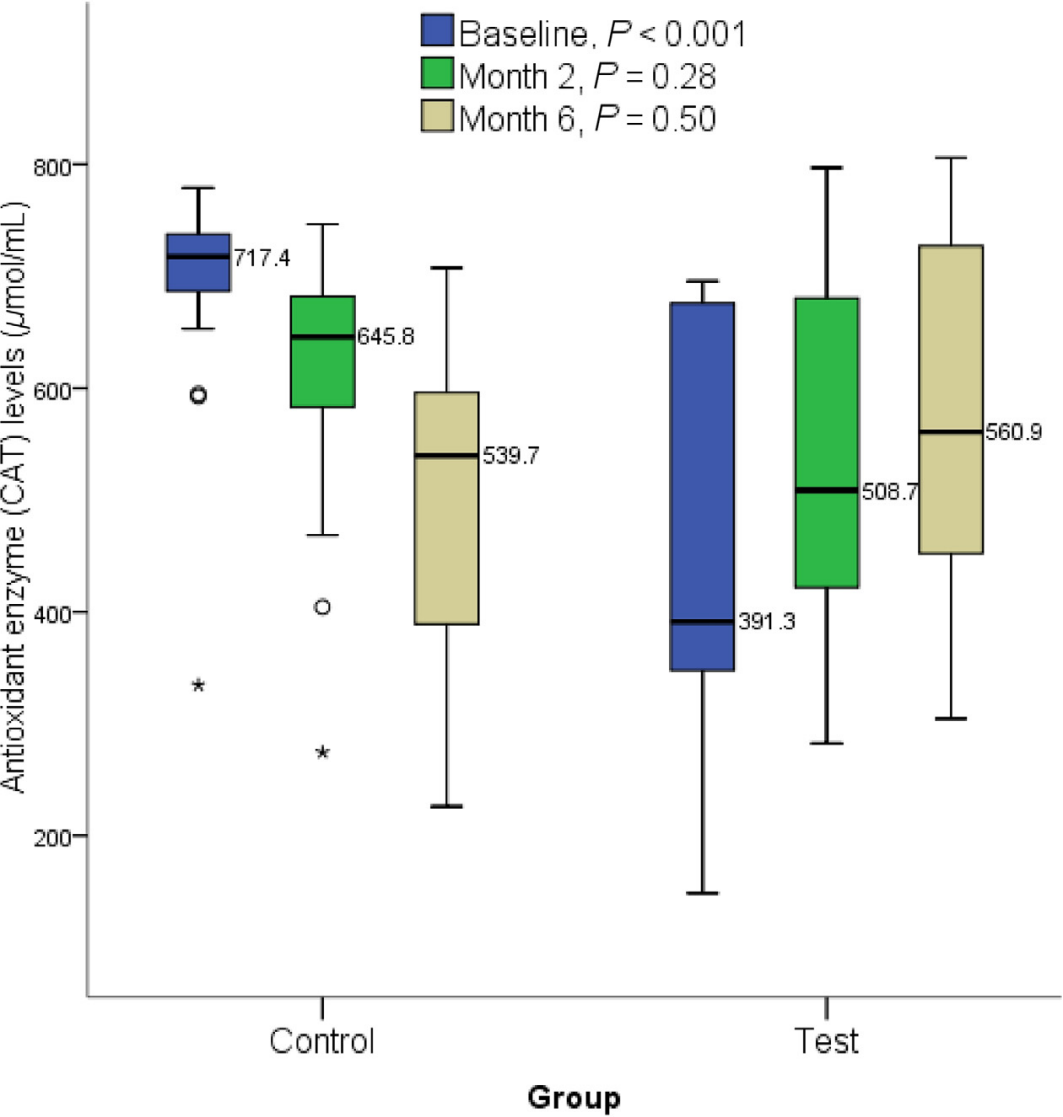
Effect of methionine and vitamin B‐complex on catalase levels at baseline, 2 and 6 months of TB treatment. Small circle “o” indicate Outliers or “out” values, while star “*” represent far out” or “Extreme values”.

**Figure 4:**
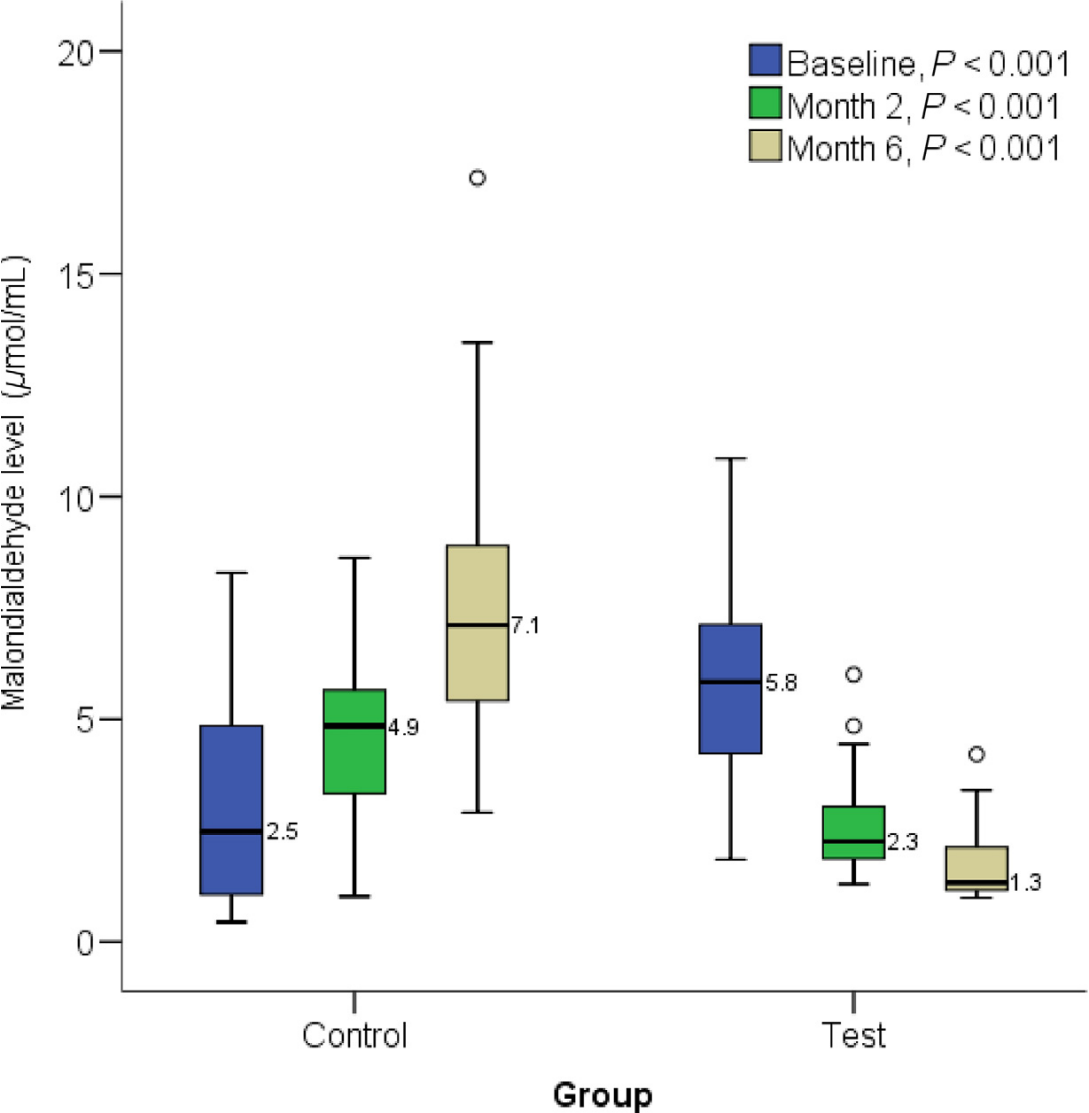
Showing effect of methionine and vitamin B‐complex on malondialdehyde levels in at 2 and 6 months of TB treatment. Small circle “o” indicate Outliers or “out” values, while star “*” represent far out” or “Extreme values”.

At the end of 2 and 6 months, GSH level was significantly (*P *=* *0.005 and *P *<* *0.001 respectively) elevated in the test group participants (median: 45.1 and 64.0) compared to the control (median: 34.7 and 20.4, respectively) (Fig. 1). At the end of 2 and 6 months, SOD level was significantly (*P *<* *0.001) elevated in the test group participants (median: 145.8 and 223.4, respectively) compared to the control (median: 68.5 and 38.2) (Fig. 2). At the end of 2 and 6 months, there was no significant difference in CAT level (*P *>* *0.05) between the test group participants and the control (Fig. 3). A significant (*P *<* *0.001) difference was observed at baseline between control (median: 717.4) and test participants (median: 391.3). At the end of 2 and 6 months, malondialdehyde level was significantly (*P *<* *0.001) lower in the test group participants (median: 2.3 and 1.3, respectively) compared to the control (median: 4.9 and 7.1, respectively) (Fig. 4).

### Effect of methionine and vitamin B‐complex on hematological indices at 2 and 6 months of antituberculosis treatment

A significant (*P *<* *0.001) difference was observed in ESR between the control and test groups at 2 months of TB treatment (Table 6). WBC count was significantly (*P *=* *0.013) lower in the test group, compared to participants in the control group. Lymphocyte count was significantly (*P *=* *0.009) lower in the test group, compared to the control group participants.

**Table 6:** Effect of methionine and vitamin B‐complex on hematological parameters at 2 months of antituberculosis treatment

	Control group a	Test group b	*P*‐value a versus b
ESR (mm/h)	45 (IQR: 35.00–60.00)	32 (IQR: 26.00–38.00)	<0.001^a^
WBC (x10^9^/L)	5.2 (IQR: 4.15–6.75)	4.41 (IQR: 3.30–5.86)	0.013^a^
RBC (x10^12^/L)	4.3 (IQR: 4.02–5.02)	4.66 (IQR: 4.13–5.12)	0.070
HGB (g/dL)	11.6 (IQR: 10.45–13.25)	12.5 (IQR: 10.90–13.60)	0.970
PCV (x10^3 ^ *μ*L)	36.2 (IQR: 33.30–40.25)	38 (IQR: 33.80–41.90)	0.117
PLT (x10^3^/L)	293 (IQR: 231.50–405.50)	275 (IQR: 211.00–380.00)	0.358
NEUT (×10^9^/L)	2.7 (IQR: 2.05–4.25)	2.34 (IQR: 1.71–3.70)	0.103
NEUT (%)	55.7 (IQR: 44.65–63.85)	52.5 (IQR: 44.10–61.90)	0.330
LYMPH (%)	33.2 (IQR: 24.85–44.45)	34.6 (IQR: 24.90–45.40)	0.825
LYMPH (×10^9^/L)	1.8 (IQR: 1.33–2.30)	1.5 (IQR: 1.20–2.05)	0.009^a^
MCV (fL)	81.4 (IQR: 76.50–89.25)	81.9 (IQR: 76.40–88.30)	0.982
MCH (pg)	26.8 (IQR: 24.50–28.70)	27.2 (IQR: 24.40–28.70)	0.993
MCHC (g/dL)	31.5 (IQR: 30.60–32.80)	31.9 (IQR: 30.40–34.00)	0.653

a Mann Whitney *U* test (Significant); IQR, Interquartile range.

There was no difference in RBC, HGB, PCV, MCV, MCH, and MCHC were higher in test group participants compared to the control group participants. PLT and neutrophil count were not different in the test versus control group participants (Table 6).

Neutrophil count was significantly (*P *<* *0.001) and PCV significantly (*P *=* *0.037) higher while there was no difference in ESR, HGB, and MCV levels in the test group participants at 6 months of treatment compared to control (Table 7). Lymphocytes (%), RBC and MCHC were significantly (*P *=* *0.008) and significantly (*P *=* *0.045 and *P *=* *0.01) lower in the test group participants at 6 months of treatment compared to control, while WBC, PLT, and MCH were not different in test versus control participants (Table 7).

**Table 7:** Effect of methionine and vitamin B‐complex on hematological parameters at 6 months of antituberculosis treatment

	Control	Test	*P*‐value
ESR (mm/h)	23 (IQR: 19.00–29.00)	32 (IQR: 23.00–41.50)	0.622
WBC (x10^9^/L)	4.05 (IQR: 3.55–5.20)	3.92 (IQR: 2.76–5.30)	0.259
RBC (x10^12^/L)	4.39 (IQR: 4.01–4.83)	4.34 (IQR: 4.08–4.82)	0.045^a^
HGB (g/dL)	12.4 (IQR: 10.83–14.15)	12.9 (IQR: 11.90–14.60)	0.798
PCV (x10^3 ^ *μ*L)	37.85 (IQR: 35.43–45.40)	38.4 (IQR: 35.00–42.20)	0.037^a^
PLT (x10^3^/L)	265 (IQR: 180.25–329.75)	228 (IQR: 190.00–304.00)	0.320
NEUT (×10^9^/L)	1.64 (IQR: 1.06–2.54)	2.29 (IQR: 1.69–4.00)	<0.001^a^
NEUT (%)	46.6 (IQR: 36.90–52.70)	55.15 (IQR: 47.08–67.23)	0.032^a^
LYMPH (%)	43.4 (IQR: 26.20–52.20)	30.65 (IQR: 23.25–42.40)	0.008^a^
LYMPH (×10^9^/L)	1.9 (IQR: 1.36–2.90)	1.32 (IQR: 0.98–2.00)	0.624
MCV (fL)	84.7 (IQR: 78.80–93.60)	88.2 (IQR: 81.25–91.30)	0.140
MCH (pg)	29 (IQR: 26.80–30.80)	27.9 (IQR: 25.75–29.80)	0.698
MCHC (g/dL)	33.2 (IQR: 31.50–34.60)	31.95 (IQR: 31.00–33.6)	0.012^a^

a Mann Whitney *U* test (significant); IQR, Interquartile range.

### Prevalence of adverse drug reactions in the different treatment groups

A significant (*P *<* *0.001) difference was observed in the treatment groups, with fewer participants developing ADRs in the test group (32.40%) compared to the control (56.20%) (Table 8). Conversely, more test group participants (67.60%) did not develop ADRs than control group participants (43.80%).

**Table 8:** Prevalence of adverse drug reactions in the treatment groups

ADR reported	All patients		Control group A		Test group b		*P*‐value a versus b
	*n*	%	*n*	%	*n*	%	
No	160	56.10	60	43.80	100	67.60	<0.001^a^
Yes	125	43.90	77	56.20	48	32.40	

a Chi‐square test (significant).

### Prevalence of different adverse drug effects between test and control groups

Comparing the test versus control, number of participants who developed generalized weakness (14. 9% vs. 35%) and tiredness (3.4% vs. 17.5%) were significantly (*P *<* *0.001) lower; rash (3.4% vs. 13.9%) and headache (2.7% vs. 12.4%) were significantly (*P *=* *0.001 and *P *=* *0.002 respectively) lower, while loss of appetite (2.7% vs. 7.3%) was significantly (*P *=* *0.099) lower in test participants compared to control (Table 9).

**Table 9:** Prevalence of different adverse drug effects between test and control groups

Type of ADR	Number (%) of patients		*P* value a versus b
	Control (a)	Test (b)	
Weakness	48 (35.00)	22 (14.90)	<0.001^a^
Tiredness	24 (17.50)	5 (3.40)	<0.001^a^
Nausea and Vomiting	42 (30.70)	35 (23.60)	0.183
Dizziness	29 (21.20)	21 (14.20)	0.122
Rash	19 (13.90)	5 (3.40)	0.001^a^
Abdominal pain	17 (12.40)	9 (6.10)	0.064
Headache	17 (12.40)	4 (2.70)	0.002^a^
Fever	8 (5.80)	4 (2.70)	0.242
Loss of appetite	10 (7.30)	4 (2.70)	0.099^a^
Dark urine	19 (13.90)	14 (9.50)	0.245
Death	1 (0.70)	0 (0.00)	0.298

a Chi‐square test (significant).

3.4% and 6.1% test group participants developed rashes and abdominal pain, respectively, compared to a higher percentage (13.9% and 12.4%) of control group subjects (Table 7). Headache, fever, and loss of appetite occurred in 2.7%, 2.7%, and 2.7% of test subjects, respectively, compared to 12.4%, 5.8%, and 7.3% in the control group subjects.

13.9% of subjects in the control group reported dark urine as opposed to 9.5% of test group subjects. Death occurred in 1 participant in the control group (Table 9).

### Association of risk factors with hepatotoxicity at 2 months of antituberculosis therapy

A significant (*P *=* *0.026) difference was observed between the control group participants (6.9%) that developed hepatotoxicity and test group participants (0%) (Table 10). A higher percentage of control (93.1%) and test (100%) did not develop hepatotoxicity. Out of the 4 participants who developed hepatotoxicity, 3 were aged 18–30 and 1 was between 31 and 40; 3 were males and 1 was a female; 1 was a smoker and 3 nonsmokers; 1 used hard drugs while 3 did not (Table 8). These risk factors (age, sex, alcohol, cigarette smoking and substance use) were not significantly associated with the development of ADRs in both control and test group participants (Table 10).

**Table 10:** Association of risk factors with hepatotoxicity at 2 months of antituberculosis therapy

Factors	Subgroup	Number (%) of patients		*P*‐value
		No hepatotoxicity	Hepatotoxicity	
Treatment Group	Control	54 (93.10)	4 (6.90)	0.026^a^
	Test	48 (100.00)	0 (0.00)	
Age (years)	18–30	28 (90.32)	3 (9.68)	0.210
	31–40	39 (97.50)	1 (2.50)	
	41–50	19 (100.00)	0 (0.00)	
	51–65	16 (100.00)	0 (0.00)	
Sex	Male	78 (96.30)	3 (3.70)	0.917
	Female	23 (95.83)	1 (4.17)	
Alcohol use	Yes	43 (97.73)	1 (2.27)	0.717
	No	55 (96.49)	2 (3.51)	
Smokes cigarette	Yes	19 (95.00)	1 (5.00)	0.773
	No	80 (96.39)	3 (3.61)	
Reported substance use	Yes	8 (88.89)	1 (11.11)	0.245
	No	90 (96.77)	3 (3.23)	

a Chi‐square test (Significant).

### Association between some risk factors and other adverse drug reactions

There was no difference observed between males than females (46.77% vs. 38.27%) who developed ADRs, and between participants in the different age groups who developed ADRs, compared to those who did not. No statistical difference was observed between overweight and obese participants (58.82% and 66.67%, respectively) developed ADRs compared to those who did not (41.18% and 33.33%) (Table 11). There was no statistical difference in weight and ADRs observed in participants who were under weight and normal weight in the test group (36.59% and 42.37%) compared to control (63.41% and 57.63%) (Table 11).

**Table 11:** Association between some risk factors and other adverse drug reactions

Risk factor	Subgroup	No ADR {*n*(%)}	ADR {*n*(%)}	*P*‐value
Sex	Male	107 (53.23)	94 (46.77)	0.194
	Female	50 (61.73)	31 (38.27)	
Age (years)	18–30	56 (57.14)	42 (42.86)	0.901
	31–40	56 (53.33)	49 (46.67)	
	41–50	26 (59.09)	18 (40.91)	
	50+	22 (57.89)	16 (42.11)	
BMI (kg/m^2^)	<18.5 (under weight)	52 (63.41)	30 (36.59)	0.293
	18.5–24.99 (normal)	68 (57.63)	50 (42.37)	
	25–29.9 (over weight)	7 (41.18)	10 (58.82)	
Alcohol intake	Yes	54 (47.37)	60 (52.63)	0.019^a^
	No	98 (61.64)	61 (38.36)	
Smokes cigarette	Yes	22 (42.31)	30 (57.69)	0.027^a^
	No	132 (59.19)	91 (40.81)	
Reported substance use	Yes	10 (41.67)	14 (58.33)	0.145
	No	140 (57.14)	105 (42.86)	

a Chi‐square test (Significant); ADR, adverse drug reaction, BMI, body mass index.

A significant (*P *=* *0.019) difference was observed amongst participants who consumed alcohol, with more of them (52.63%) developing ADRs compared to those that did not take alcohol (47.37%) (Table 11). Fewer people 38.36% that did not consume alcohol developed ADRs than those who did not develop any ADR. A significant (*P *=* *0.027) difference was observed amongst participants who consumed smoked, with more of them (57.63%) developing ADRs compared to those that did not smoke (42.31%) (Table 11).

More participants who reported using hard drugs (58.33%) developed ADRs compared to those that did not use any hard drug (41.67%). 42. 86% of participants that did not use any hard drug developed ADRs, compared to those that did not develop any ADR (57.14%) (Table 11).

### Logistic regression for factors influencing development of adverse drug reactions

The factors identified to be significantly associated with Adverse Drug Reaction in bivariate analysis (Table 11) were harvested and subjected to multivariate analysis. The dependent variable in Table 12 above is Adverse Drug Reaction status, a Yes‐or‐No outcome. Patients who take alcohol were about two times more likely (OR = 1.501, *P *=* *0.173, 95% CI: 1.02, 3.3) to develop Adverse Drug Reaction than those who do not take alcohol. Also, patients who smoke have about forty percent increase in risk of developing Adverse Drug Reactions (OR = 1.371, *P *=* *0.402, 95% CI: 0.656, 2.864) compared to those who do not smoke. The model was a good fit as Hosmer–Lemeshow goodness of fit was not significant (*χ*2 = 1.220, *P *=* *0.543) (Table 12).

**Table 12:** Logistic regression for factors influencing development of adverse drug reactions

Variables	Odds ratio	SE	Wald statistic	*P*‐value	95% CI
Alcohol intake					
Yes	1.501	0.298	1.856	0.173	(0.837, 2.692)
No^a^					
Smoking status					
Yes	1.371	0.376	0.704	0.402	(0.656, 2.864)
No^a^					

a Reference category.

### Association between some risk factors and ADR by treatment group

More control group participants who consumed alcohol (75. 47%) and smoked cigarettes (78.57%) developed ADRs in a significant (*P *=* *0.000) and significant (*P *=* *0.010) manner compared to those who did not smoke (51.46%) or consume alcohol (44.16%). Participants in the control group who used hard drugs (73.33%) developed ADRs in an insignificant (*P *>* *0.05) manner compared to those who did not (Table 13).

**Table 13:** Association between some risk factors and ADR by treatment group

Characteristic		Control			Test		
		No ADR *n*(%)	ADR *n*(%)	*P* value	No ADR *n*(%)	ADR *n*(%)	*P* value
Alcohol intake	Yes	13 (24.53)	40 (75.47)	0.000^a^	41 (67.21)	20 (32.79)	0.945
	No	43 (55.84)	34 (44.16)		54 (66.67)	27 (33.33)	
Smokes cigarette	Yes	6 (21.43)	22 (78.57)	0.010^a^	16 (66.67)	8 (33.33)	0.893
	No	50 (48.54)	53 (51.46)		81 (68.07)	38 (31.93)	
Reported substance use	Yes	4 (26.67)	11 (73.33)	0.195	6 (66.67)	3 (33.33)	0.937
	No	50 (44.25)	63 (55.75)		89 (67.94)	42 (32.06)	

a Chi‐square test (Significant).

More test group participants who consumed alcohol (67.21%), smoked cigarettes (66.67%) and used hard drugs (66.67%) did not develop ADRs in an insignificant (*P *>* *0.05) manner compared to those who developed ADRs (32.79%, 33.33% and 33.33%, respectively)(Table 13).

## Discussion

Hepatic transaminases, renal, hematological and antioxidant indices are mostly affected by deleterious antitubercular drug‐induced toxicity in patients undergoing treatment. An elevation in AST and the liver‐specific ALT would, therefore, indicate leakage from injured tissues (Ozer et al. 2008), while an increase in ALP level occurs due to overproduction and release in blood (Ramaiah 2007). Supplementation with agents capable of modulating the harmful effects of these antitubercular medicines would be of immense benefit to patients undergoing treatment for tuberculosis. Achieving this would reduce often severe adverse effects, enhance compliance and ultimately improve treatment outcomes. Compared to participants on only the antitubercular medicines, administration of methionine and vitamin B‐complex (in the presence of antitubercular medicines) showed a significant (*P *<* *0.001) decrease in total bilirubin and the liver enzymes‐ALT, AST, and ALP at the end of the intensive phase of treatment. This decrease can be attributed to the reported antioxidant activity of methionine and B‐complex, while the higher levels in the control group support the knowledge that antitubercular medicines, particularly rifampicin, induce hepatocellular injury and hyperbilirubinemia (Singh et al. 2016).

Tuberculosis and the liver are related in many ways, one of which is the direct hepatic involvement by the disease itself that can impair hepatic functions (Essop et al. 1984) and elevate indices like ALT, AST, and ALP. It is thus possible to infer that as the bacterial load reduced in the course of treatment (from the 2nd to the 6th month of treatment), the toxic effect of the infection reduced, allowing the hepatic and renal cells to recover and reduce elevated parameters. This is important, considering that the liver is a regenerative organ. According to the standard protocol of tuberculosis treatment in Nigeria, pyrazinamide and rifampicin (a known nephrotoxic (Manika et al. 2013) and hepatotoxic (Awodele et al. 2011)) agent, are not included in the continuation phase of treatment, as opposed to their use in the intensive phase (along with isoniazid and ethambutol). Thus, the assault on the liver is thus less, therefore improving the ability of the hepatic cells to regenerate. These reasons are likely responsible for recovery of many of the liver and renal function parameters with time in the control group.

Previous studies indicate a strong association between renal injury and oxidative stress in patients treated with antituberculosis drugs (Kwon et al. 2004; Schubert et al. 2010). This causes an elevation in creatinine, urea (Yanardag et al. 2005) and albumin and a decrease in total protein levels (Shabana et al. 2012). This correlates to results from this study when baseline values are compared to values at the end of 2 months of therapy in the treatment groups. Comparing the treatment groups at the end of 2 months showed a significant (*P *<* *0.001) decrease in urea and creatinine and a significant (*P *=* *0.096) increase in albumin in participants co‐administered methionine and vitamin B‐complex with the combined antitubercular medicines, indicating an amelioration of the deleterious effects of antitubercular agents. These parameters were similarly affected up to the end of 6 months of therapy but in an insignificant (*P *>* *0.05) manner. Albumin has been reported to possess antioxidant properties (Roche et al. 2008), which makes it possible to postulate a potentiation of the antioxidant potential of albumin and methionine/vitamin B‐complex for this effect. In this present study, total protein was higher in the control group at the end of 6 months compared to test group participants, possibly due to higher globulin fraction relative to albumin fraction. This corresponds to the result of Shingdang et al. (2016), which showed higher globulin levels compared to albumin at the end of the continuation phase of TB treatment. Levels of globulin were, however, not evaluated in this present study.

Hematological changes associated with tuberculosis treatments have been reported in many parts of the world (Kassa et al. 2016). Results from this current study showed lower levels of RBC, HGB, PCV, and PLT in control group subjects at the end of the intensive phase of treatment, relative to test subjects. This suggests antitubercular drug‐induced deleterious effects on these indices, for example, isoniazid, which has been reported to cause a decrease in HGB synthesis (Ghosh et al. 2017). Methionine and vitamin B‐complex are therefore able to inhibit this effect by promoting HGB synthesis, hence its increase in test group subjects. PCV was observed in the present study to be higher in patients on combined antitubercular medicines, methionine and vitamin B‐complex, relative to those on antitubercular medicines alone. This is similar to the results of Bharti et al. (2017), who reported that propolis, an antioxidant, elevated both HGB and PCV following exposure to combined antitubercular medicines. It is reasonable to, therefore, state that methionine, which has been reported to have free radical scavenging activity, would produce a similar effect.

A previous study had reported that INH‐induced oxidative stress in red blood cells (RBCs) (Yilmaz et al. 2008), and also inhibits hem biosynthesis (Huang and Benz 2001). This corresponds to results from this present study, where the RBC counts were lower in participants on antitubercular medicines compared to those on methionine and vitamin B‐complex (in the presence of antiTB medicines). This modulatory role can be explained by the link between phosphatidylcholine, methionine and vitamin B12. Phosphatidylcholine, a major component of red blood cell membranes (Cooper and Hausman 2015), is synthesized from the metabolite of methionine, S‐Adenosyl‐L‐Methionine (SAMe). Vitamin B12 is used by methionine synthase to convert homocysteine into methionine (Seetharam and Li 2000). This is further converted to S‐Adenosyl‐L‐Methionine, which in turn synthesizes phosphatidyl choline. Administering methionine and vitamin B‐complex to TB patients on anti‐TB medicines would, therefore, help stabilize red blood cells leading to higher counts.

Amilo et al. (2013) stated that white blood cells (WBC) count tend to be higher among TB patients, compared to normal health patients, with neutrophils being implicated as the main contributor to the increase. During TB treatment, the bacterial load is expected to decrease as the antitubercular medicines clear the *Mycobacterium tuberculosis*. The white blood cells that would be required to fight the infection would therefore decrease. This observation was made in the present research. The WBC count was lower in patients taking methionine and vitamin B‐complex (in the presence of anti‐TB medicines), relative to patients administered the anti‐TB medicines alone. Tuberculosis may cause neutropenia (Koju et al. 2005), which corresponds to results of the present study. Exposure to methionine and vitamin B‐complex further decreased neutrophil, further confirming their modulatory role in antitubercular medicine induced toxicity.

Platelets, involved in the inflammatory and immunological response, have the capacity to release cytokines and chemokines, thus acting as immune regulators (Trzeciak‐Ryczek et al. 2013). The direct relationship between platelet and WBC is logical because when there is an immune response due to TB infection, platelets tend to increase. It is thus reasonable to state that treatment, which would see a reduction in the TB infective state, would result in a decrease in WBC and platelet counts.

Elevated ESR, an indicator of disease severity, was observed in our study at baseline. This correlates with other studies that also reported elevated ESR in tuberculosis patients (Hungund et al. 2012). At 2 months of commencing treatment, the modulatory effect of methionine and vitamin B‐complex reduced ESR levels in a significant (*P *<* *0.001) manner, compared to patients on only the antitubercular medicines.

Oxidative stress caused by antitubercular medicines leads to the depletion of glutathione, the major endogenous antioxidant (Stine and Chalasani 2015), while isoniazid depletes vitamin B6 stores (Burda et al. 2007). As a result, methionine, a precursor of glutathione and vitamin B6 need to be supplemented from external sources. Vitamin B‐complex tablets serve as a source of vitamin B6 and vitamin B12 that is vital in the Methionine‐Homocysteine‐Folate‐B12 Cycle (Seetharam and Li 2000).

Antitubercular medicines like isoniazid produce toxic metabolites, acetyl isoniazid, and hydrazine, which inactivate catalase and superoxide dismutase to induce oxidative stress (Zhai et al. 2008). Following an injury to the liver caused by antitubercular medicines, endogenous antioxidant enzymes (CAT, SOD), and the nonenzymatic antioxidant glutathione (GSH) decrease while malondialdehyde (MDA), a marker of lipid peroxidation, increases (Liu et al. 2017); corresponding to results from the current research. However, in the test subjects, methionine and vitamin B‐complex co‐administered with the combination antitubercular medicines corrected these abnormalities compared to patients on only the antitubercular medicines. This modulation of antioxidant activity was obvious by a significant (*P *<* *0.001) increase in GSH and SOD and a significant (*P *<* *0.001) decrease in lipid peroxidation, similar to that reported by Anisimova et al. (2013). This observation was at the end of the intensive and continuation phases of treatment. The antioxidant activity of S‐Adenosyl‐L‐Methionine (SAMe) (a metabolite of methionine) is mainly due to its role as a precursor of GSH, the major endogenous antioxidant (Niedzwiecki et al. 2013). SAMe was found to be able to prevent and reverse hepatotoxicity associated with several drugs and increase GSH levels (Anstee and Day 2012).

Toxicity due to antitubercular medicines can manifest clinically as adverse effects (Blumberg et al. 2003). A significant (*P *<* *0.001) decrease in the prevalence of ADRs in test group participants (56.2%) was observed in the present research, compared to control (32.4%). Rash was reported in 3.4% of patients co‐administered antitubercular medicines with methionine and vitamin B‐complex, compared to 13.9% on only antitubercular medicines. INH, RIF, PZA, and EMB have been reported to cause this adverse effect in tuberculosis patients (Kaswala 2013).

Males tend to engage more in smoking, use of alcohol consumption than females; all these are risk factors that increase their susceptibility to tuberculosis (Jeong et al. 2015). This could also be responsible for the observation in the present research where more males (46.77%) developed adverse drug reactions (ADRs) than females (38.27%). A significant interaction occurred between smoking (*P *=* *0.027), alcohol use (*P *=* *0.019) and adverse drug reactions in the present study, with smokers having about forty‐percent increase in risk of developing adverse drug reactions, compared to those who do not smoke, while patients who take alcohol regularly were about two times more likely to develop ADRs than those who do not take alcohol. This link between smoking, alcohol and adverse drug effects was similarly reported by (Chung‐Delgado et al. 2011).

In this present research, hepatotoxicity was observed in 6.9% of control group participants on only the antitubercular medicines, lower than 18.2% reported by Isa et al. (2016). Hepatotoxicity is caused by isoniazid and rifampicin in patients (Devarbhavi et al. 2010). One death was reported in the present study due to complications of tuberculosis and possibly, drug‐induced hepatotoxicity as a secondary cause.

## Conclusion

Toxicity to hepatic, renal, and hematological indices and the antioxidant system, as well as adverse effects observed in patients exposed to antitubercular medicines during the 6‐month period of treatment was modulated by the combination of methionine and vitamin B‐complex tablets. This clearly indicates that such interventions could form part of new treatment strategies aimed at reducing adverse effects due to the antitubercular medicines and improve treatment outcomes in tuberculosis patients.

## Disclosure

The authors declare that no conflict of interest exists in the course of conducting and funding this research. All authors had final decision regarding the manuscript and the decision to submit the findings for publication.
